# Immune Checkpoint Blockade Improves Chemotherapy in the PyMT Mammary Carcinoma Mouse Model

**DOI:** 10.3389/fonc.2020.01771

**Published:** 2020-09-10

**Authors:** Evelyn Sirait-Fischer, Catherine Olesch, Annika F. Fink, Matthias Berkefeld, Arnaud Huard, Tobias Schmid, Kazuhiko Takeda, Bernhard Brüne, Andreas Weigert

**Affiliations:** ^1^Faculty of Medicine, Institute of Biochemistry I, Goethe-University Frankfurt, Frankfurt, Germany; ^2^Research Center of Oncology, ONO Pharmaceutical Co., LTD, Osaka, Japan; ^3^Frankfurt Cancer Institute, Goethe-University Frankfurt, Frankfurt, Germany; ^4^German Cancer Consortium (DKTK), Partner Site Frankfurt, Frankfurt, Germany; ^5^Branch for Translational Medicine and Pharmacology TMP of the Fraunhofer Institute for Molecular Biology and Applied Ecology IME, Frankfurt, Germany

**Keywords:** cancer, immunotherapy, chemotherapy, immune checkpoint, cytotoxic lymphocytes

## Abstract

Despite the success of immune checkpoint blockade in cancer, the number of patients that benefit from this revolutionary treatment option remains low. Therefore, efforts are being undertaken to sensitize tumors for immune checkpoint blockade, which includes combining immune checkpoint blocking agents such as anti-PD-1 antibodies with standard of care treatments. Here we report that a combination of chemotherapy (doxorubicin) and immune checkpoint blockade (anti-PD-1 antibodies) induces superior tumor control compared to chemotherapy and immune checkpoint blockade alone in the murine autochthonous polyoma middle T oncogene-driven (PyMT) mammary tumor model. Using whole transcriptome analysis, we identified a set of genes that were upregulated specifically upon chemoimmunotherapy. This gene signature and, more specifically, a condensed four-gene signature predicted favorable survival of human mammary carcinoma patients in the METABRIC cohort. Moreover, PyMT tumors treated with chemoimmunotherapy contained higher levels of cytotoxic lymphocytes, particularly natural killer cells (NK cells). Gene set enrichment analysis and bead-based ELISA measurements revealed increased IL-27 production and signaling in PyMT tumors upon chemoimmunotherapy. Moreover, IL-27 signaling improved NK cell cytotoxicity against PyMT cells *in vitro*. Taken together, our data support recent clinical observations indicating a benefit of chemoimmunotherapy compared to monotherapy in breast cancer and suggest potential underlying mechanisms.

## Introduction

The idea to engage the immune system in the fight against cancer was already proposed in the early twentieth century but was then mainly disregarded (1). Over a century later, the discovery of immune checkpoints as brakes of the immune system and the possibility to unleash those brakes to fight cancer was rewarded with the Nobel Prize in Physiology in 2018 as a new principle for cancer immunotherapy (Press release: The Nobel Prize in Physiology or Medicine 2018). Allison, Honjo and their coworkers discovered and elucidated the function of the negative costimulatory molecules cytotoxic T lymphocyte–associated protein 4 (CTLA-4) and programmed death 1 (PD-1), respectively (2, 3). The blockade of those inhibitory checkpoint receptors by neutralizing monoclonal antibodies is now well-known as immune checkpoint blockade and is already broadly used in the clinic. Since the first clinical trial using immune checkpoint inhibitors in 2000, there have been numerous clinical trials with either anti-CTLA-4 or anti-PD-1 as single agent drugs. To date there have been at least 500 clinical studies with PD-1 blockers conducted on at least 20 cancer types (4). Anti-PD-1 drugs are now approved for a variety of highly immunogenic cancer types, including non-small cell lung cancer, renal cell carcinoma, hodgkin's lymphoma, and metastatic melanoma. Remarkably, PD-1 blockade has shown positive results in all mentioned malignancies, measured by the overall response rate (5). However, a significant proportion of patients does not respond to immunotherapy (6). Indeed, in patients with metastatic breast cancer, single-drug anti-PD-1 therapy has shown little efficacy, due to a lower mutational load and a lower abundance of tumor-infiltrating lymphocytes (TILs) (7). Therefore, new strategies are needed to enhance the efficacy of anti-PD-1 treatment in breast cancer. In the last few years, approaches to combine PD-1 blockade with conventional treatments such as chemotherapy have shown promising results even as first-line treatment in triple-negative metastatic breast cancer [(8), NCT02425891]. It is important to note, that chemotherapy still represents the preferred standard of systemic treatment for metastatic breast cancer and remains one of the most efficient ways to improve patient outcome by decreasing tumor burden and metastasis (9). However, major limitations of chemotherapy remain, foremost non-specific toxicity, and tumor chemoresistance (10, 11). Interestingly, a recent study suggested an involvement of PD-1 signaling in the acquisition of chemoresistance and therefore emphasized the rationale for a combinatorial chemoimmunotherapy in the clinical setting (12). In addition, chemotherapy was also shown to increase the immune infiltrate and inhibit immunosuppressive components in the tumor microenvironment, which in turn can improve immune checkpoint blockade. Taken together, these findings substantiate combinatorial chemoimmunotherapy as a reasonable approach to fight breast cancer. In this study, we therefore analyzed the impact of combinatorial chemotherapy and immune checkpoint blockade in the PyMT mammary carcinoma mouse model (13), since previous studies using this model failed to show effectiveness of anti-PD-1 monotherapy (14, 15).

## Materials and Methods

### Animal Experiments

Female mice expressing the polyoma virus middle T oncoprotein (PyMT) under the Mouse Mammary Tumor Virus (MMTV) promoter in a C57BL/6 background were used. In the PyMT model, mice spontaneously develop tumors in each mammary gland starting from 8 weeks after birth. Mice were divided into four groups according to treatment (anti-PD-1, IgG1, doxorubicin (DOX) + anti-PD-1, and DOX + IgG1). For animals receiving immune checkpoint blockade only, treatment was initiated (day 0) once the first tumor reached a size of 0.6 cm in diameter. Antibodies were administrated i.p. at a concentration of 20 mg/kg (on day 0) and 10 mg/kg (on day 6, 12, 18). All mice received either anti-mouse PD-1 antibody (4H2, Ono Pharmaceutical, Osaka, Japan) or anti-mouse IgG1 (BioXcell/Hölzel Diagnostik, Cologne, Germany) diluted in sterile 0.9% NaCl. In the model with the combination of chemotherapy and immune checkpoint blockade treatment started once the first mammary tumor reached a size of 1 cm in diameter. Doxorubicin (Cell Pharm, Bad-Vilbel, Germany) diluted in sterile 0.9% NaCl was administrated i.p. (5 mg/kg) once a week for 5 weeks. One day after doxorubicin administration, mice were treated with 10 mg/kg of either anti-mouse PD-1 antibody (4H2, Ono Pharmaceutical) or anti-mouse IgG1 (BioXCell). Mice were monitored three times a week for up to 5 weeks after initial treatment. Tumor size was determined by tumor palpating. The tumor volume was calculated using the formula: V = length × width^2^ × π/6. For all animal experiments the guidelines of the Hessian animal care and use committee were followed (approval numbers: FU1127, FU1191).

### Flow Cytometry

Tumor single cell suspensions were generated using the Tumor Dissociation Kit and the gentleMACS™ Dissociator (both from Miltenyi Biotec, Bergisch Gladbach, Germany) using standard protocols. The following anti-mouse antibodies were used for staining of single cell suspensions: anti-CD3-PE-CF594, anti-CD4-BV711, anti-CD8-BV650, anti-CD11c-BV711, anti-CD19-APC-Cy7, anti-CD45-AlexaFluor700, anti-CD49f-PE-CF594, anti-CD146-AlexaFluor488, anti-CD326-BV711, anti-Ly6C-PerCP-Cy5.5, anti-NK1.1-BV510 (all from BD Biosciences, Heidelberg, Germany), anti-CD31-PE-Cy7, anti-CD117-APC-eFluor780 (both from eBioscience, San Diego, USA), anti-CD90.2-PE, anti-MHC-II-APC (both from Miltenyi Biotec), anti-CD11b-BV605, anti-CD324-AlexaFluor647, anti-F4/80-PE-Cy7, anti-GITR-FITC, anti-Ly6G-APC-Cy7, anti-SiglecH-FITC, and anti-γδTCR-APC (all from Biolegend, San Diego, USA). NK/PyMT cell co-culture samples were stained with the following antibodies. anti-CD25-PE-Cy7, anti-CD69-BV605, anti-CD107a-PE and anti-NK1.1-APC (all from Biolegend). Samples were acquired with a LSR II/Fortessa™ flow cytometer (BD Biosciences) and analyzed using FlowJo software V10 (BD Biosciences). All antibodies and secondary reagents were titrated to determine optimal concentrations. CompBeads (BD Bioscience) were used for single-color compensation to create multi-color compensation matrices. For gating, fluorescence minus one (FMO) controls were used. The instrument calibration was controlled daily using Cytometer Setup and Tracking (CS&T) beads (BD Bioscience).

### RNA Sequencing

Total RNA was isolated from snap frozen PyMT tumors using the peqGOLD Total RNA Kit (VWR International, Darmstadt, Germany). RNA samples were analyzed on a 2100 Bioanalyzer using Agilent RNA 6000 Nano chip (both from Agilent Technologies, Santa Clara, USA). Library preparation was performed using the SMARTer® Stranded Total RNA Sample Prep Kit–HI (Takara Bio Europe, Saint-Germain-en-Laye, France). Quantity and quality of the cDNA libraries were determined by Qubit™ dsDNA HS Assay Kit (Thermo Fisher Scientific, Dreieich, Germany) and Agilent High Sensitivity DNA chip (Agilent Technologies). Libraries were sequenced on a NextSeq 500 sequencer (single end, 75 cycles) using V2 chemistry (Illumina, San Diego, USA). Sequencing data were analyzed using the SeqBox software (16). In brief, after adapter trimming with skewer (17), the software used STAR (18) to map the reads to the mouse reference genome (mm10) and RSEM (19) for gene and isoform-level quantification, which allows the differential expression analysis by DESeq2 (20).

### Analysis of Publicly Available Human Mammary Carcinoma Datasets

The METABRIC data set (21) was used to determine patient survival according to the gene signatures obtained from the PyMT mouse model upon combinatorial chemoimmunotherapy.

### Phenoptics™ Immunofluorescence Analysis

Tumors were zinc-fixed, paraffin-embedded, and subsequently stained in a fluorescent multiplex immunohistochemistry staining using the Opal^TM^ 7-Color Fluorescent Immunohistochemistry (IHC) Kits (Akoya, Marlborough, USA). The following anti-mouse antibodies were used: anti-αSMA (Sigma-Aldrich, Schnelldorf, Germany F3777), anti-DIO2 (Elabscience, Houston, USA, E-A-13198), anti-GSN (Biozol, Eching, Germany, BOB-PA2109), anti-MMP3 (Santa Cruz, Heidelberg, Germany, sc-21732), anti-Pan-Cytokeratin (Abcam, ab27988), anti-PD-L1 (Cell signaling, D5V38), and anti-PDK4 (Antibodies-online, Aachen, Germany, ABIN3028963) in an automated staining using the BOND RX Automated IHC Research Stainer (Leica Biosystems, Nussloch, Germany). Stained tumor sections were scanned using Vectra® 3 automated quantitative pathology imaging system and analyzed using inForm® software V2.3 (both Akoya). Marker expression in the cytoplasm was quantified with the inForm® software using a positivity or 4-bin (0–3+) scoring algorithm (22). For the latter spectrally unmixed fluorescence signals in the cytoplasm of epithelial or stromal cells were grouped into four bins based on signal distribution (0 = lowest signal, 3 = highest signal), indicating differences in protein expression.

### Gene Set Enrichment Analysis

Using gene expression values (expression >0.1 log2 TPM values after DESeq2) between individual treatment groups as an input, enriched biological processes were identified using Gene Set Enrichment Analysis (GSEA) version 4.0.0 (23).

### Protein Quantification

Tumor interstitial fluids were obtained by manual cryopulverization and subsequent incubation with 1:2 tumor weight/volume of 2 × PBS for 3 h at 4°C under rotation. The LEGENDplex™ mouse inflammation panel (Biolegend) was used to determine cytokines levels in the tumor supernatants. To quantify protein levels in NK/PyMT cell co-culture supernatants, ELISA kit for PRF1 (Abbexa, Cambridge, UK, abx258736) as well as the mouse IFN-γ Flex Set (BD Bioscience, 558296) were utilized according to the manufacturer's instructions. Bead-based array samples were acquired by flow cytometry and analyzed using FlowJo V10.

### Cytotoxicity Assay

NK cells were isolated from spleens of either wildtype (WT) or IL-27 receptor α (IL-27Rα) KO mice using the EasySep™ Mouse NK Cell Isolation Kit (STEMCELL™ Technologies, Vancouver, Canada). NK cells used as effector cells were co-cultured for 4 h at 37°C with PyMT target cells at different effector cell-target cell ratios, as indicated. Both NK cells and PyMT cells were labeled with different fluorescent dyes (PKH67 & PKH26, Sigma-Aldrich) and dead PyMT cells were identified using 7-AAD staining (Miltenyi Biotec). Living (7-AAD-negative) PyMT cells were subsequently determined via flow cytometry.

### Quantitative PCR

RNA was isolated as described above followed by cDNA transcription using the Sensiscript® cDNA synthesis kit (Qiagen, Hilden, Germany). The following murine primers were used: *Cd25*, sense: 5′-CAAGAACGGCACCATCCTAAA-3′, anti-sense: 5′-TCCTAAGCAACGCATATAGACCA-3′; *Cd69*, sense: 5′-AAGCGATATTCTGGTG AACTGG-3′, anti-sense: 5′-ATTTGCCCATTTCCATGTCTGA-3′; *Prf1*, sense: 5′-CTG CCACTCGGTCAGAATG-3′, anti-sense: 5′-CGGAGGGTAGTCACATCCAT-3′. *Rps27a* served as internal control. Data were analyzed using QuantStudio™ (Thermo Fisher Scientific).

### Statistics

Data are presented as means ± SEM. Statistical comparisons between two groups were performed using either two-way ANOVA, Mann-Whitney test or unpaired two-tailed Student's *t*-test as indicated. For the latter two data were pre-analyzed to determine normal distribution and equal variance with D'Agostino–Pearson omnibus normality test. Differences in patient survival were analyzed using Log-rank (Mantel–Cox) test. Statistical analysis was performed with GraphPad Prism V8. Differences were considered significant at *p* < 0.05. Asterisks indicate significant differences between experimental groups (^*^p < 0.05, ^**^p < 0.01, ^***^p < 0.001, ^****^p < 0.0001).

## Results

### Doxorubicin Chemotherapy Improves the Response to PD-1 Blockade

**Figure 1:**
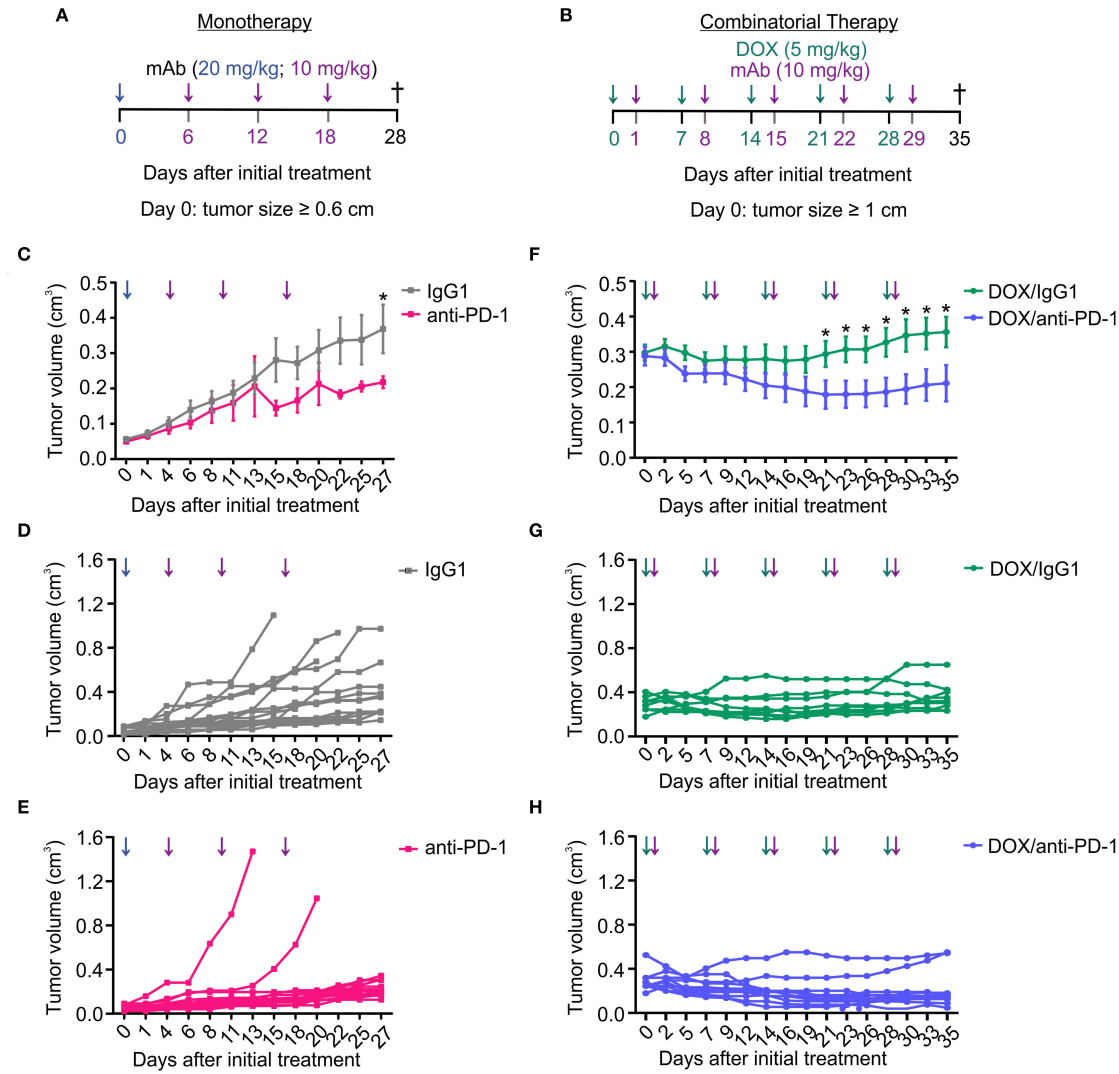
Combination of chemotherapy and PD-1 blockade improves tumor control in the PyMT model. Treatment regimens for anti-PD-1 monotherapy and doxorubicin (DOX) plus anti-PD-1 combinatorial therapy. **(A)** Treatment regimen of monotherapy. PyMT tumors were treated with either anti-PD-1 or isotype control (IgG1) antibody (i.p.) every 6 days for 18 days (day 0 = 20 mg/kg; day 6, 12, 18 = 10 mg/kg) once the first tumor reached a size of 0.6 cm in diameter. **(B)** Treatment regimen of combinatorial therapy. PyMT tumors were treated with 5 mg/kg doxorubicin (DOX) (i.p.) and with 10 mg/kg of either anti-PD-1 or isotype control (IgG1) antibody (i.p.) once weekly for 5 weeks once the first tumor reached a size of 1 cm in diameter. **(C,F)** Cumulative tumor volume (length × width^2^ × π/6) of primary tumors upon **(C)** monotherapy (*n* = 17 each) and **(F)** combinatorial therapy (DOX/IgG1: *n* = 11, DOX/anti-PD-1: *n* = 10) over time are shown, as well as the individual tumor volumes for **(D,E)** monotherapy and **(G,H)** chemoimmunotherapy. Data are means ± SEM, *p*-values were calculated using unpaired *t*-test; *p < 0.05.

We and others previously observed that anti-PD1 therapy was poorly effective in the PyMT mouse model of mammary carcinoma (14, 15). Sensitizing non-responsive tumors for immune checkpoint blockade is a major goal in current immunotherapy. Therefore, we asked whether a combinatorial approach consisting of doxorubicin (DOX) chemotherapy and anti-PD-1 antibody administration has an enhanced efficacy in reducing tumor growth compared to anti-PD-1 monotherapy. Tumors in the PyMT mouse model arise spontaneously starting 8 weeks after birth. A therapeutic setting was employed, where treatment was initiated once a tumor diameter of 0.6 cm (anti-PD-1 alone) or 1 cm (DOX/anti-PD-1) had been reached. The smaller initial size in case of anti-PD-1 monotherapy was chosen to allow monitoring tumor growth over 4 weeks without reaching ethical endpoints of tumor size. Mice received intraperitoneal (i.p.) injections with either a PD-1-blocking antibody (10–20 mg/kg) or an IgG1 isotype control antibody (10–20 mg/kg) alone or with preceding DOX administration i.p. (5 mg/kg) (Figures 1A,B). Although anti-PD-1 monotherapy significantly slowed progression of primary tumors compared to the IgG1 control, this effect was modest, and we did not observe tumor regression (Figures 1C–E). In contrast, combinatorial therapy with DOX and anti-PD-1 antibody not only markedly suppressed tumor progression but also significantly reduced tumor volumes from day 21 onwards when compared to the DOX/IgG1 control (Figures 1F–H). Although tumor reduction was also observed upon DOX/IgG1 administration at least in some tumors, the majority of DOX/IgG1 treated tumors either responded poorly or relapsed toward the end of the study (Figure 1G). Notably, only two DOX/anti-PD-1 mice showed tumor progression (Figure 1H). In conclusion, these results show that, in the PyMT tumor model, the efficacy of anti-PD-1 treatment is enhanced by DOX chemotherapy as indicated by a partial tumor remission upon combinatorial chemoimmunotherapy.

### Increased NK Cell Infiltrate Upon Combinatorial Chemotherapy and PD-1 Blockade

**Figure 2:**
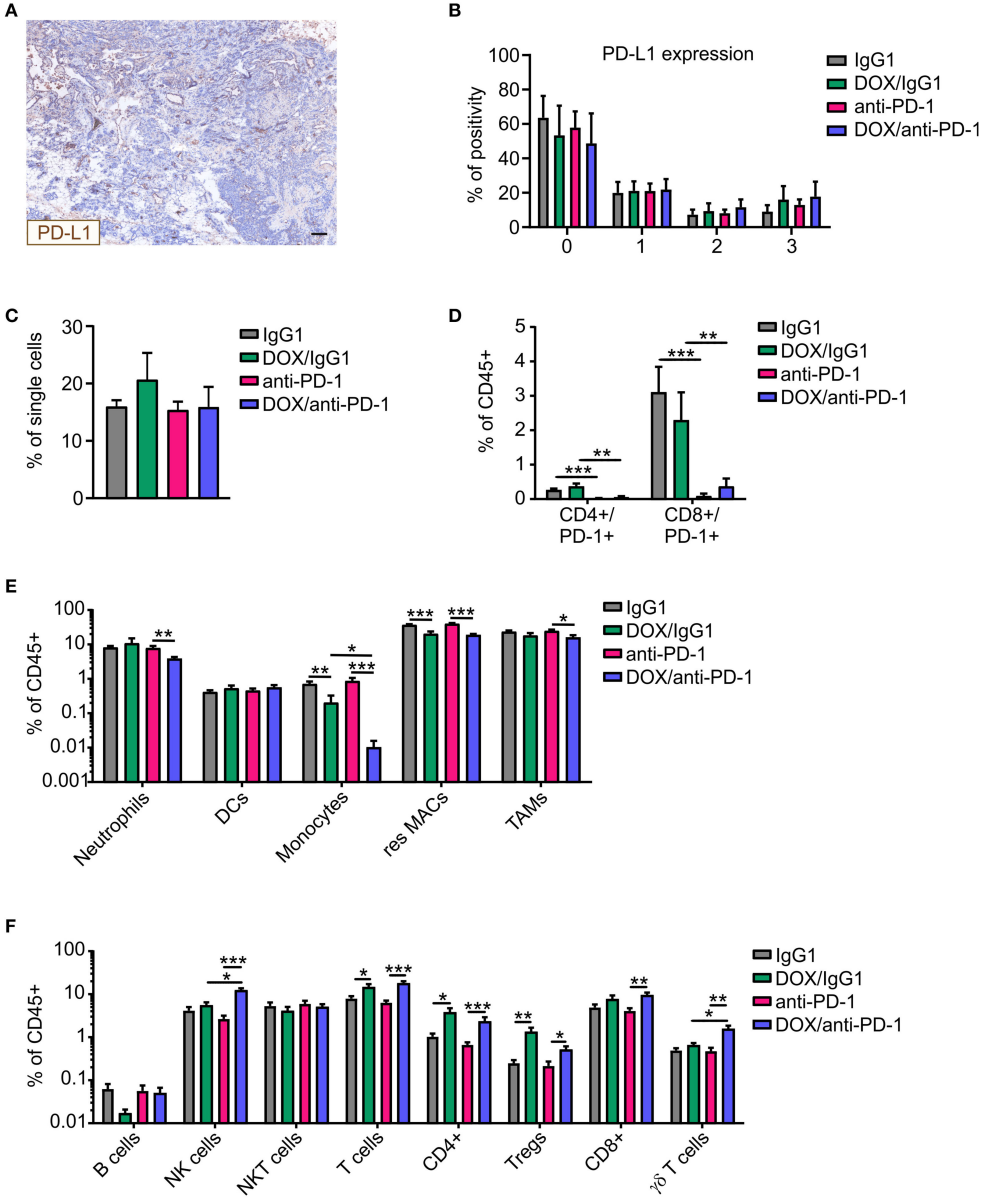
Altered immune cell composition in PyMT tumors upon chemoimmunotherapy. PyMT tumor-bearing animals were treated with either anti-PD-1 or doxorubicin (DOX) plus anti-PD-1 and the corresponding IgG1 antibody control. PyMT tumor sections (*n* ≥ 4) were stained for PD-L1 as well as for DAPI (nuclei) and analyzed using Phenoptics™. **(A)** Representative image shows PD-L1 expression for a doxorubicin (DOX) plus anti-PD-1 section. Scale bar: 100 μm. **(B)** Quantification of PD-L1 percentage positivity using the inForm® software with a 4-bin scoring algorithm (0, lowest expression; 3, highest expression). The relative frequencies of **(C)** CD45+ immune cells within total single cells and **(D)** CD4+/PD-1+ and CD8+/PD-1+ T cells **(E)** myeloid cell subsets as well as **(F)** lymphoid cell subsets relative to total CD45+ immune cells are displayed (IgG1: *n* = 17, anti-PD-1: *n* = 16, DOX/IgG1: *n* = 10, DOX/anti-PD-1: *n* = 9). Data are means ± SEM, *p*-values were calculated using unpaired *t*-test or Mann–Whitney test according to D'Agostino & Pearson omnibus normality test; *p < 0.05, **p < 0.01, ***p < 0.001.

We wondered whether the increased susceptibility to chemoimmunotherapy was associated with increases in PD-L1 expression in tumors upon chemotherapy. Therefore, PyMT tumor sections of all four treatment groups were stained for PD-L1 and DAPI (nuclei) using Phenoptics™ multiplex IHC staining (Figure 2A). Interestingly, PD-L1 expression scoring with the inForm® software using a 4-bin scoring algorithm revealed no alteration in PD-L1 expression in tumors of the different treatment regimens (Figure 2B). Thus, alterations in PD-L1 expression did not account for improved tumor control due to chemoimmunotherapy. Next, multicolor flow cytometry analysis of tumor single-cell suspensions of all four treatment groups was performed at the experimental endpoint to investigate cellular alterations potentially increasing efficacy of the combinatorial therapy (Supplementary Figure 1). Flow cytometry revealed no differences in CD45+ immune cell abundance between the different treatments (Figure 2C). Administration of the neutralizing anti-PD-1 antibody induced an efficient depletion of PD-1 on CD4+ and CD8+ T cells as compared to the corresponding IgG1 control, while chemotherapy *per se* did not alter the abundance of PD-1-expressing T cells within the total immune cell population (Figure 2D). Within the CD45+ immune cell population, dendritic cell (DC) levels were unchanged, whereas monocyte and resident macrophage abundance was reduced upon chemotherapy. For monocytes, this reduction was even accentuated when the anti-PD1 antibody was applied (fold change monocytes: IgG1:DOX/IgG1 = 0.32, anti-PD-1:DOX/anti-PD-1 = 0.012; resident macrophages: IgG1:DOX/IgG1 = 0.55, anti-PD-1:DOX/anti-PD-1 = 0.47) (Figure 2E). Furthermore, neutrophil and tumor-associated macrophage (TAM) frequencies decreased after DOX/anti-PD-1 administration compared to anti-PD-1 monotherapy (fold change neutrophils: 0.49; TAMs: 0.65). Although B cell and NKT cell numbers in the lymphoid cell lineage were unaltered, overall T cell levels including CD4+ T cell and Treg frequencies increased after chemotherapy (fold change T cells: IgG1:DOX/IgG1 = 1.79, anti-PD-1:DOX/anti-PD-1 = 2.77; CD4+: IgG1:DOX/IgG1 = 3.62, anti-PD-1:DOX/anti-PD-1 = 3.43; Tregs: IgG1:DOX/IgG1 = 5.24, anti-PD-1:DOX/anti-PD-1 = 2.35) (Figure 2F). Moreover, chemotherapy in combination with anti-PD-1 administration enhanced CD8+ T abundance compared to anti-PD-1 monotherapy by 2.2-fold. Most interestingly, γδ T cell and NK cell levels were elevated upon combinatorial DOX/anti-PD-1 therapy as compared to monotherapy or DOX/IgG1 administration (fold change γδ T cells: anti-PD-1:DOX/anti-PD-1 = 3.22, DOX/IgG1:DOX/anti-PD-1 = 2.4; NK cells: anti-PD-1:DOX/anti-PD-1 = 4.54, DOX/IgG1:DOX/anti-PD-1 = 2.24). Taken together, flow cytometry data did not provide a clear explanation on the cellular mechanisms responsible for the increased efficacy of chemoimmunotherapy. However, increased cytotoxic lymphocyte levels, including NK cells, upon combinatorial DOX/anti-PD-1 therapy emerged as a promising lead.

### Gene Signatures Predict Survival of Human Mammary Carcinoma Patients

**Figure 3:**
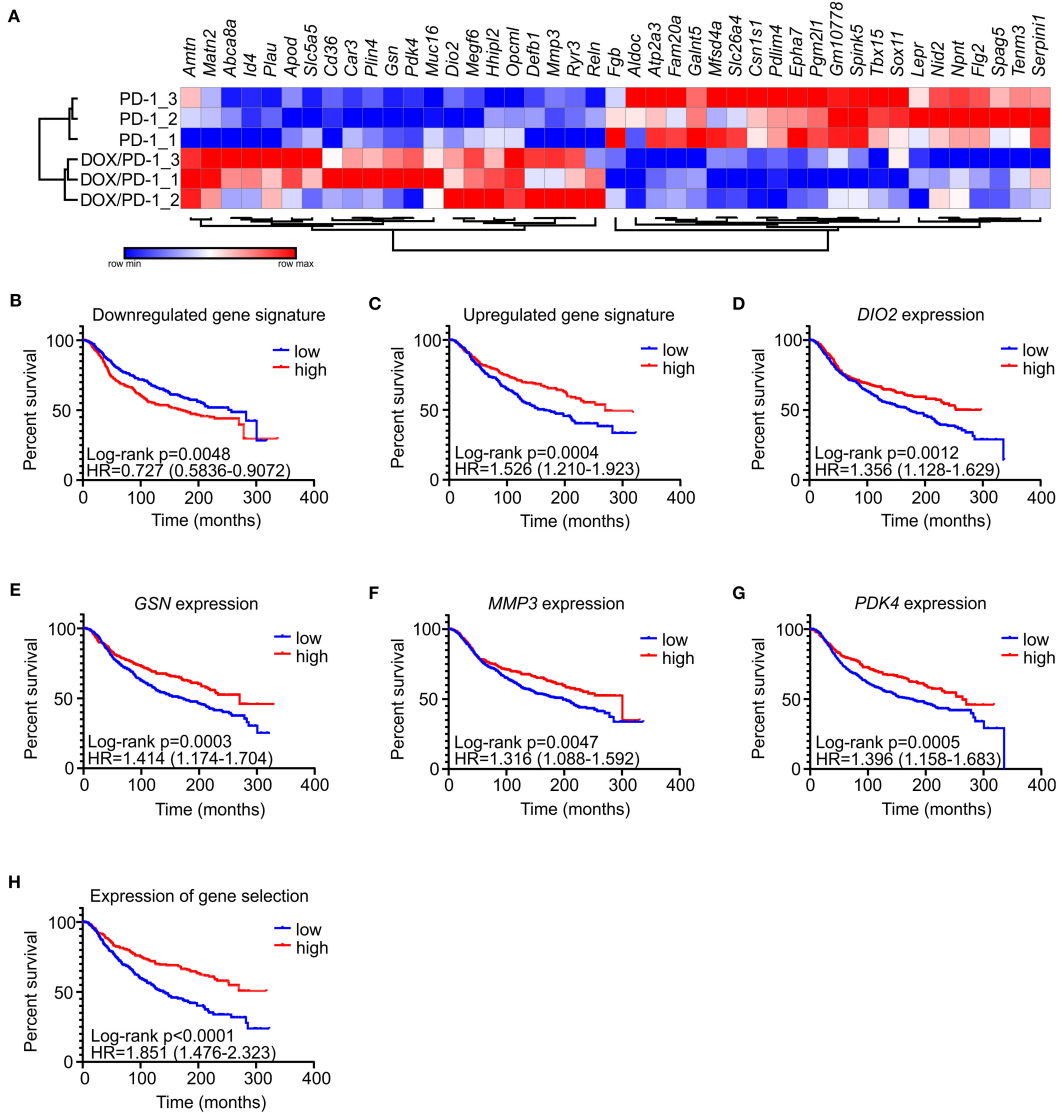
Gene signatures of PyMT tumors treated with DOX/anti-PD-1 combinatorial therapy predict human mammary carcinoma patient survival. **(A)** Comparative transcriptome analysis of PyMT tumors upon anti-PD-1 treatment and doxorubicin (DOX) plus anti-PD-1 therapy (*n* = 3 each). Transcriptomes were generated by RNA seq. The heat map shows differentially expressed genes between both groups. **(B–H)** The METABRIC dataset (21) was analyzed for a correlation with gene signatures derived from PyMT tumors treated with DOX/anti-PD-1 compared to anti-PD-1 monotherapy. Patients were grouped into quartiles based on unranked mean expression of up- or downregulated genes and survival was analyzed. Survival rates of patients expressing high (>75% percentile) or low (<25% percentile) levels of the signature genes were compared. Shown are survival rates of patients expressing the gene set that was **(B)** downregulated or **(C)** upregulated upon DOX/anti-PD-1 therapy. The survival rates of patients expressing individual predictive upregulated genes **(D–G)**, or mean expression of these genes **(H)** are displayed. *p*-values were calculated using log-rank test.

To gain explanations for increased NK cell frequencies upon chemoimmunotherapy and to gain further insights into potential other mechanisms explaining the success of DOX/anti-PD-1 combinatorial therapy vs. monotherapy, whole transcriptome RNA-Seq was performed. For this purpose, mRNA was isolated from whole PyMT tumors, sequenced using NextSeq 500 and data were analyzed using DESeq2 (differentially regulated genes: adjusted *p* < 0.1; log_2_ fold change in expression >1). Only 4 genes were found to be significantly altered between the IgG1 control and the anti-PD-1 monotherapy group, and 19 genes were altered when comparing the DOX/IgG1 with the DOX/anti-PD-1 group, while 93 genes were differently regulated between the DOX/IgG1 and the IgG1 group (Supplementary Table 1, Supplementary Figure 2). 43 genes were found to be differentially expressed, comparing anti-PD-1 treatment to the combination of DOX and anti-PD-1 (Figure 3A). There was no meaningful overlap between the different gene signatures (Supplementary Figure 3), indicating that each treatment group was characterized by a unique response pattern. Out of the 43 genes altered when comparing anti-PD-1 to chemoimmunotherapy, 21 were upregulated upon DOX/anti-PD-1 administration relative to anti-PD-1 monotherapy, whereas 22 were downregulated. To test the validity of these gene signatures, we analyzed if they would hold predictive value in human mammary carcinoma. Therefore, mean expression values of genes either up- or downregulated in our model were obtained from the METABRIC data set (21). These mean expression values were then compared with clinical data in the same dataset (Figures 3B–H). Patients were grouped into quartiles based on the unranked mean expression of the different gene signatures and survival rates of patients with low expression (<25% percentile) were compared to those with high expression (>75% percentile). Strikingly, analyzing the METABRIC dataset revealed that patients expressing low levels of genes downregulated in PyMT tumors treated with chemoimmunotherapy showed improved survival (Figure 3B). This was even more pronounced for patients expressing high levels of genes that were upregulated in PyMT tumors treated with combinatorial therapy (Figure 3C). Hence, patient prognosis improved if they showed high expression of genes that were upregulated upon DOX/anti-PD-1 treatment and inversely also improved if they showed low expression levels of genes that were downregulated upon DOX/anti-PD-1 treatment in the PyMT model. Since the difference in patient survival was more notable when using the upregulated gene signature, all upregulated genes were further analyzed on their individual impact on patient survival in the METABRIC dataset. Amongst all upregulated genes, four genes were found to be individually associated with improved patient survival, namely type II iodothyronine deiodinase (*DIO2*), gelsolin (*GSN*), matrix metalloproteinase 3 (*MMP3*) and pyruvate dehydrogenase kinase 4 (*PDK4*) (Figures 3D–G). Accordingly, a gene signature consisting of these four genes more accurately discriminated patients with improved or reduced survival prognosis when compared to the gene signature of all 21 upregulated genes (Figure 3H). DIO2 processes the hormone thyroxine (T4) to the more potent triiodothyronine (T3) to enhance growth, development and metabolism (24). DIO2 was overexpressed in brain tumors (oligoastrocytoma, glioblastoma, oligodendroglioma, pituitary tumors) and in thyroid adenoma (24), and in endometrial and colorectal cancer high expression was associated with a favorable prognosis (25). PDK4 regulates glucose metabolism and mitochondrial respiration and can have oncogenic or tumor suppressive effects depending on cancer type. In hepatocellular carcinoma downregulation of PDK4 is associated with poor prognosis (26), and PDK4 downregulation in lung cancer promoted cell proliferation and tumor growth (27), while high PDK4 expression was correlated with poor patient outcome in breast cancer (28). GSN and MMP3 are both involved in extracellular matrix (ECM) remodeling. GSN is a ubiquitous actin filament-severing protein (29), whose tumor-suppressive functions on various cancer types when highly expressed were previously noted (30, 31). In colon cancer, for instance, overexpression of GSN reduces proliferation and invasion of colon carcinoma cells (32) and in breast cancer downregulation of GSN correlates with malignant progression (33). MMP3 degrades several components of the ECM. Previous studies attributed oncogenic effects to MMP3 (34, 35), and high expression of MMP3 is considered unfavorable in pancreatic, pulmonary, and mammary carcinoma (36).

### Histological Validation of Predictive Genes Confirms Transcriptome Analyses

**Figure 4:**
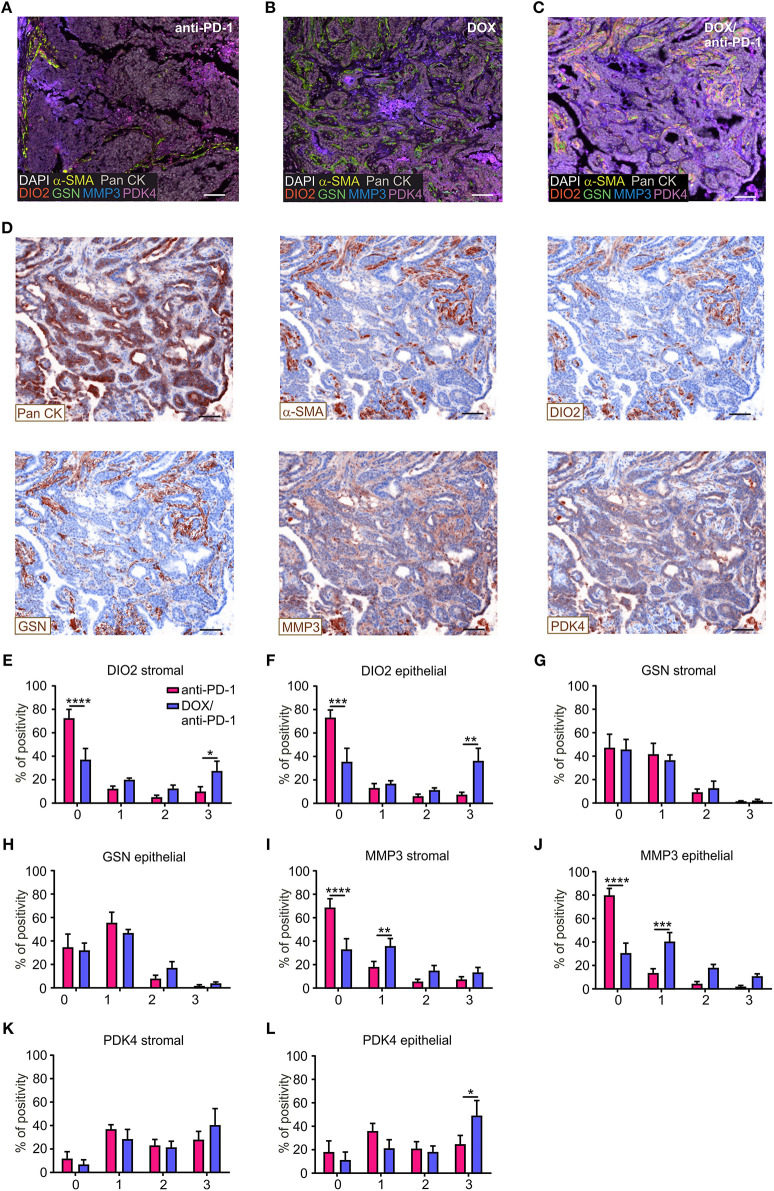
Histological validation of predictive genes. PyMT tumor sections (*n* = 6 each) were stained for DIO2, GSN, MMP3, PDK4, Pan-Cytokeratin (Pan CK; epithelial marker), α-SMA (stromal marker), DAPI (nuclei), and analyzed using Phenoptics™. Representative images show combined expression of all markers for **(A)** anti-PD-1, **(B)** doxorubicin (DOX), and **(C)** DOX plus anti-PD-1 treated tumors as well as **(D)** the expression of single markers for the DOX/anti-PD-1 section. Scale bars: 100 μm. **(E–L)** Quantification of marker percentage positivity using the inForm® software and a 4-bin scoring algorithm (0, lowest expression; 3, highest expression). Data are means ± SEM, *p*-values were calculated using two-way ANOVA with uncorrected Fisher's LSD test, *p < 0.05, **p < 0.01, ***p < 0.001, ****p < 0.0001.

To validate the impact of these four selected target genes upon chemoimmunotherapy at protein level, PyMT tumor sections of all four treatment groups were stained using Phenoptics™ multiplex IHC staining. Therefore, tumor sections were stained for the four specific prognostic markers, as well as for Pan-Cytokeratin (Pan CK) as an epithelial/tumor marker, alpha-smooth muscle actin (α-SMA) as a stromal marker and were counterstained with DAPI (Figures 4A–D). Tumor tissues were segmented into stromal and epithelial compartments and the four markers were quantified within these two tumor fractions, respectively, using the inForm® software with a 4-bin scoring algorithm (Figures 4E–L). Spectrally unmixed fluorescence signals in the cytoplasm of epithelial or stromal cells were grouped into four bins based on signal distribution (0 = lowest signal, 3 = highest signal), indicating differences in protein expression. The distribution within the four bins was calculated accordingly. These analyses revealed that DIO2 expression was significantly elevated in both the epithelial and the stromal compartment of DOX/anti-PD-1 treated tumors compared to anti-PD-1 only treated tumors, as represented by decreased levels in the first bin (lowest expression) and enhanced levels in the fourth bin (highest expression) (Figures 4E,F). In contrast, GSN expression was unchanged throughout the different treatments and bins (Figures 4G,H). MMP3 expression decreased in the chemoimmunotherapy group in the first bin in both the stromal and epithelial compartment (Figures 4I,J), indicating that tumors administered with DOX/anti-PD-1 showed enhanced protein levels of MMP3 as compared to anti-PD-1 treated tumors. Finally, PDK4 signals were solely increased in the epithelial section of tumors treated with DOX/anti-PD-1 combination therapy (Figures 4K,L). Overall, the histology data generally supported our findings at the transcriptome level, since three out of four markers that were transcriptionally upregulated upon chemoimmunotherapy, and were predictive in human mammary carcinoma patients were also elevated at protein level. The individual function of these proteins in the context of tumor control remains to be determined.

### IL-27 Is Induced Upon Chemoimmunotherapy and Enhances NK Cell Activation and Cytotoxicity Toward PyMT Tumor Cells

**Figure 5:**
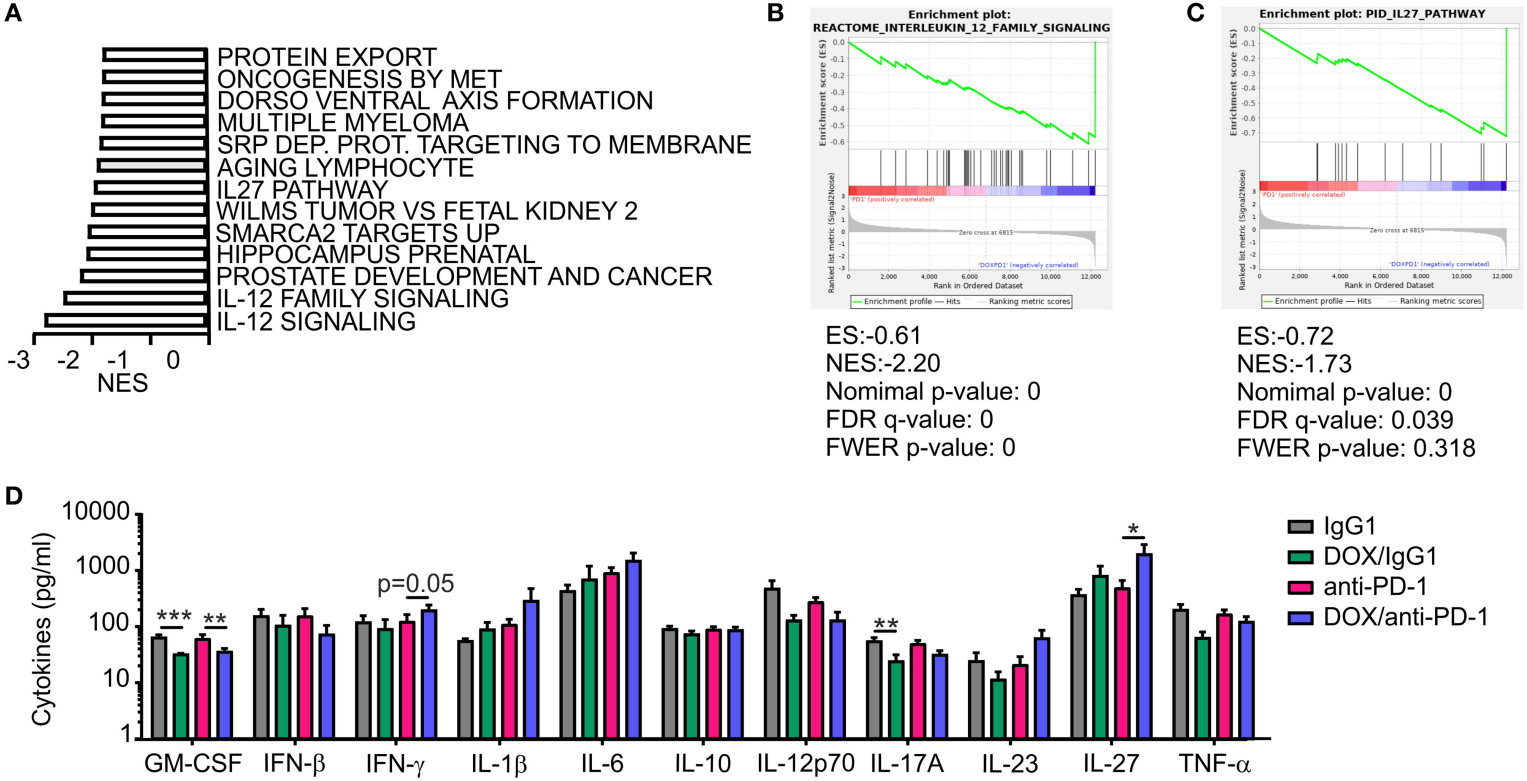
Increased IL-27 expression in PyMT tumors upon DOX/anti-PD-1 therapy. **(A–C)** Gene set enrichment analysis (GSEA) was performed using transcriptome data of tumors from PyMT mice treated with doxorubicin (DOX) plus anti-PD-1 compared to anti-PD-1 monotherapy. **(A)** Significantly (*p* < 0.05; false discovery rate (FDR) <0.25) enriched pathways upon DOX/anti-PD-1 therapy compared to anti-PD-1 monotherapy are shown. NES, normalized enrichment score. Selected enrichment plots for **(B)** IL-12 family signaling and **(C)** IL-27 pathway are displayed. ES, enrichment score; FDR, false discovery rate; FWER, familywise error rate; NES, normalized enrichment score. **(D)** Quantification of cytokine levels in PyMT tumors upon DOX/anti-PD-1 or anti-PD-1 monotherapy and the corresponding IgG1 controls using LEGENDplex™ are displayed. Data are means ± SEM, *p*-values were calculated using unpaired *t*-test or Mann–Whitney test according to D'Agostino & Pearson omnibus normality test, *p < 0.05, **p < 0.01, ***p < 0.001.

**Figure 6:**
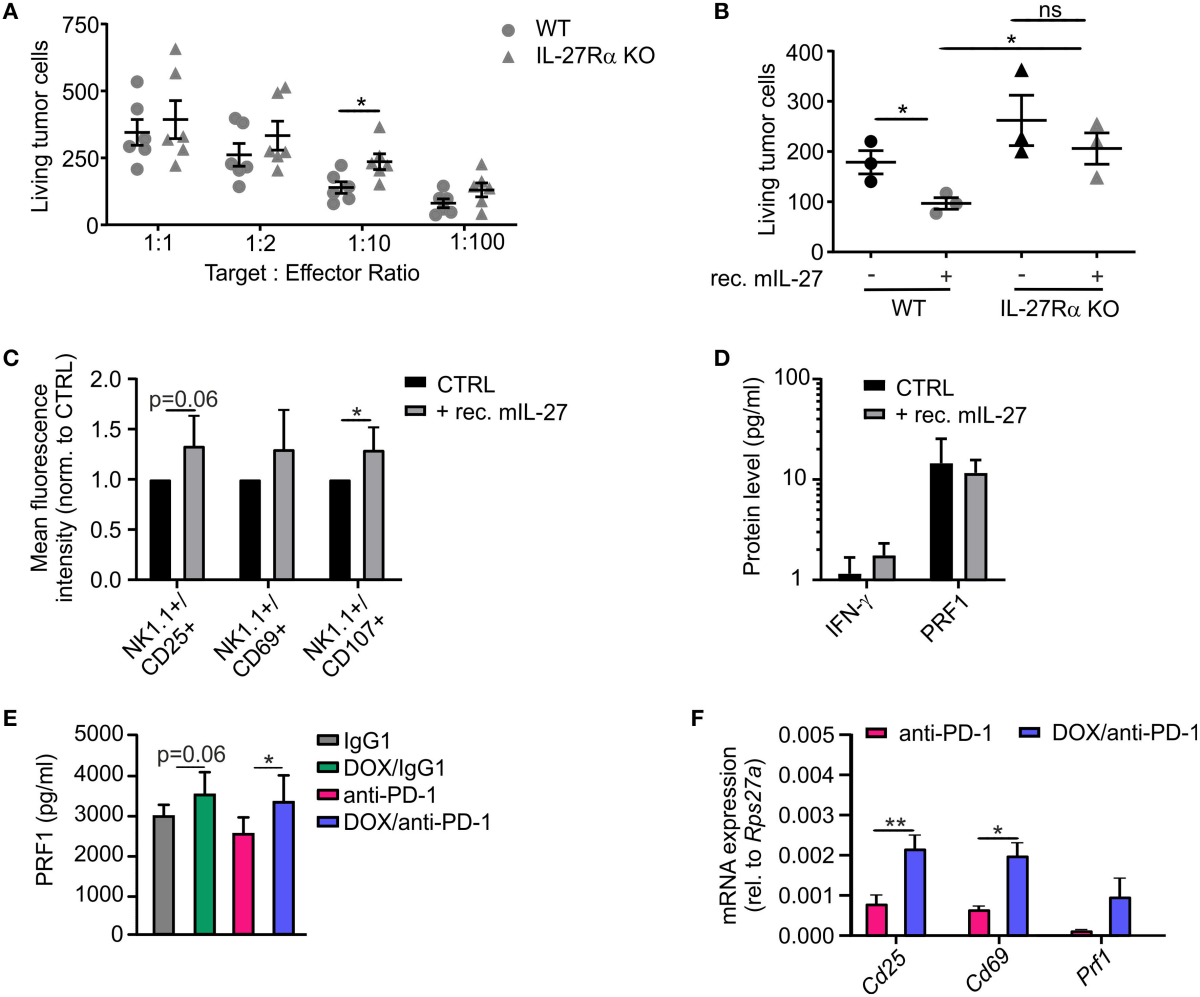
IL-27 improves NK cell activation and cytotoxicity toward PyMT cells. **(A,B)** NK cells were isolated from spleens of wildtype (WT) or IL-27 receptor α KO (IL-27Rα KO) mice and co-cultured with PyMT tumor cells for 4 h at 37°C. Afterwards, living PyMT cells were determined by flow cytometry using 7-AAD staining. PyMT tumor cell viability **(A)** dependent on addition of WT or IL-27Rα KO NK cells at different target: effector ratios (*n* = 6 each) and **(B)** at a target: effector ratio of 1:10 with or without addition of 20 ng/ml recombinant murine IL-27 (*n* = 3 each) are shown. **(C,D)** NK cells were isolated from WT spleens and co-cultured with PyMT tumor cells for 4 h at 37°C at a target: effector ratio of 1:10 with or without (CTLR) addition of 20 ng/ml recombinant murine IL-27 (*n* = 5 each). **(C)** NK cells were subsequently stained for expression of NK1.1, CD25, CD69, and CD107 and analyzed by flow cytometry. The mean fluorescence intensities normalized to the CTRL are shown. **(D)** Quantification of NK activation markers on protein level in the co-culture supernatants is displayed. **(E,F)** mRNA and interstitial fluid were extracted from whole PyMT tumors of all treatment regimens. **(E)** Quantification of PRF1 protein levels in interstitial fluid and **(F)** NK activation markers on mRNA level is shown. Data are means ± SEM, *p*-values were calculated using unpaired *t*-test or Mann–Whitney test according to D'Agostino & Pearson omnibus normality test or one sample *t*-test, *p < 0.05, **p < 0.01. N.d., not detectable; ns, not significant.

In addition to the histological analysis, transcriptome data were also used for gene set enrichment analyses (GSEA) to identify gene sets that were differentially regulated between individual treatment groups within the Molecular Signatures Database (normalized enrichment score ≥1.6, *p* ≤ 0.05, FDR *q* ≤ 0.25). DOX monotherapy (+ IgG1) induced the most prominent changes (58 gene sets induced) when compared to IgG1 treatment alone, with a number of pathways being induced by DOX treatment that suggest changes in intracellular signaling events (Supplementary Table 2). When performing GSEA to compare pathways between the DOX/anti-PD-1 and the anti-PD-1 group, we found that 13 gene sets enriched upon (Figure 5A; Supplementary Table 2). Amongst the gene sets most significantly enriched in the DOX/anti-PD-1 group were IL-12 family signaling, as well as individual pathways within this cytokine family, namely IL-12 and IL-27 signaling (Figures 5B,C). These GSEA results raised the question whether IL-12 or IL-27 protein levels were altered in PyMT tumors when comparing chemoimmunotherapy and anti-PD-1 monotherapy. Therefore, tumor interstitial fluids of all initial four treatment groups were analyzed via the LEGENDplex™ Mouse Inflammation Panel, determining protein levels of 13 different cytokines (Figure 5D). While most cytokine levels were not significantly altered, chemotherapy reduced IL-17A levels as well as GM-CSF levels. However, most interestingly, whereas IL-12p70 amounts were rather, although not significantly, decreased, IL-27 levels were elevated upon chemotherapy plus anti-PD-1 treatment compared to anti-PD-1 monotherapy. Since these data suggested an involvement of IL-27 signaling in the anti-tumor efficacy of chemoimmunotherapy and flow cytometry analysis revealed enhanced NK cells frequencies upon this combinatorial treatment, we wondered whether IL-27 would directly affect NK cell cytotoxicity. Therefore, we performed a NK cell cytotoxicity assay using NK cells from spleens of either wild type (WT) or IL-27 receptor α (IL-27Rα) KO mice as effector cells that were co-cultured with PyMT target cells at different effector cell-target cell ratios. NK cells and PyMT cells were labeled with different fluorescent dyes and live vs. dead PyMT cells were identified by 7-AAD staining. Analyzing tumor cell viability in the cytotoxicity assay demonstrated a significantly decreased cytotoxicity of NK cells derived from IL-27Ra KO mice toward PyMT tumor cells at a target cell-effector cell ratio of 1:10 when compared to the WT NK cells (Figure 6A). At other ratios, no significant differences in cytotoxicity were observed. To further explore the effect of IL-27 on NK cell cytotoxicity, the assay was repeated at the 1:10 ratio, with or without the addition of 20 ng/ml recombinant murine IL-27. The data again indicated a decreased cytotoxicity of IL-27Ra KO NK cells toward PyMT tumor cells and, more importantly, revealed an enhanced cytotoxicity of WT NK cells, but not IL-27Ra KO NK cells, when supplemented with recombinant IL-27 (Figure 6B). These data suggest that IL-27 produced upon chemoimmunotherapy has the capacity to increase NK cell cytotoxicity toward PyMT tumor cells. Next, we asked how IL-27 may improve NK cell effector functions. To elucidate this, the NK cell cytotoxicity was repeated using WT NK cells and PyMT cells in co-culture without (CTRL) or supplemented with 20 ng/ml recombinant murine IL-27. Afterwards, the co-cultured cells were stained for NK1.1, CD25, CD69, and CD107a to determine their activation status (Figure 6C). Flow cytometry analysis indeed revealed tendencies for increased mean fluorescence intensities (MFI) upon IL-27 addition for all three activation markers, reaching significance for CD107. To further characterize NK cell effector functions, the protein levels for the NK cell-derived cytolytic protein perforin (PRF1) as well as for IFN-γ as another activation marker were quantified in the co-culture supernatants (Figure 6D). While IFN-γ was not detectable, PRF1 protein levels did not differ significantly upon IL-27 supplementation. These data suggest that PRF1 release *per se* was not the driver of IL-27-dependent NK cell activation in the *in vitro* assay. However, PRF1 levels were also determined in PyMT tumor interstitial fluids of all treatment groups (Figure 6E). This analysis revealed that DOX administration in general enhanced PRF1 amounts, which was significant upon DOX/anti-PD-1 treatment as opposed to anti-PD-1 monotherapy. Next, the mRNA expression levels of *Cd25, Cd69*, and *Prf1* in whole tumors of both anti-PD-1 groups were analyzed (Figure 6F). Although mRNA expression of *Prf1* was not significantly changed, a tendency for elevated levels was found, corresponding to protein data (Figure 6E). Notably, mRNA expression of both activation markers *Cd25* and *Cd69* was increased in tumors treated with chemoimmunotherapy as compared to those treated with anti-PD-1 alone. While these molecular alterations in PyMT tumors cannot be attributed exclusively to NK cells, they support a milieu containing activated lymphocytes upon chemoimmunotherapy in PyMT tumors compared to anti-PD-1 monotherapy.

## Discussion

While immune checkpoint blockade such as anti-PD-1 and anti-PD-L1 treatment proved to be impressively effective across a wide range of cancer types (37–39) only a small fraction of breast cancer patients benefits from anti-PD-(L)1 monotherapy (5, 40). Consequently, an obvious approach to improve response rates is the combination of immune checkpoint blockade and standard regimens such as chemotherapy. Indeed, our study showed an enhanced efficacy of anti-PD-1 administration plus DOX chemotherapy in reducing the growth in PyMT tumors compared to monotherapy. In line with our findings, preclinical studies demonstrated the efficacy of anti-PD-(L)1 plus different chemotherapy agents in murine colon and lung adenocarcinoma models (41, 42). Interestingly, a recent clinical phase 3 study (IMpassion130) assessing the efficacy and safety of atezolizumab (anti-PD-L1 antibody) plus nab-paclitaxel (chemotherapy) in patients with unresectable, locally advanced or metastatic triple-negative breast cancer (TNBC) reported a clinically meaningful overall survival benefit with chemoimmunotherapy in patients with PD-L1 immune cell-positive disease (43). These findings are supported by another recent phase 2 clinical trial (TONIC trial) (44). Patients suffering from metastatic TNBC were treated with nivolumab (anti-PD-1 antibody) without or with additional irradiation, cyclophosphamide, cisplatin, or DOX treatment. In this cohort, the objective response rate was highest in patients treated with nivolumab in combination with chemotherapy, particularly with DOX (44). This was attributed to the induction of T cell cytotoxicity pathways and an inflammatory gene signature including JAK-STAT and TNF-α signaling after DOX treatment in responders. Our data confirm the potential advantage of DOX in combination with anti-PD-1 treatment. This may extend beyond TNBC since the PyMT model is considered closely resembling the situation in human HER2-positive mammary tumors. Moreover, our RNA-Seq approach identified genes related to IL-12/IL-27 signaling, which also includes JAK-STAT pathway genes and molecules involved in triggering cytotoxic lymphocytes, which is another similarity to the TONIC trial.

In an attempt to identify the immune cell subsets that could have mediated the anti-tumor effect upon DOX/anti-PD-1 treatment, we detected elevated levels of NK cells when comparing PyMT mice receiving DOX/anti-PD-1 treatment to mice receiving monotherapy. CD8+ T cells and γδ T cells were elevated in the DOX/anti-PD-1 group compared to the group receiving anti-PD-1 as single agent, again indicating a sensitizing effect of chemotherapy. These lymphocyte subsets are known for their ability to effectively kill tumor cells (45, 46). We focused on NK cells given their specific induction only in the combination therapy group. It has been shown that the cytolytic functions of NK cells can be markedly improved by immune checkpoint blockade or chemotherapy (47, 48). We observed an involvement of IL-27 signaling in the more efficient chemoimmunotherapy compared to monotherapy. Importantly, we were able to demonstrate an IL-27-dependent higher cytotoxicity of NK cells toward PyMT tumor cells. Supporting our results, previous studies have identified IL-27 as an NK cell activator by promoting their cell viability and cytolytic activity in several cancer models (49). Moreover, IL-27 has been shown to enhance the activation and proliferation of CD8+ T cells (50) and to trigger anti-tumor functions in γδ T cells (51), thus also affecting T cell subsets that were elevated upon DOX/anti-PD-1 treatment in our study. Taken together, our data suggest an involvement of IL-27 and cytotoxic lymphocytes such as NK cells in the efficacy of chemoimmunotherapy in the PyMT model. An individual contribution of these immune cell subsets may be tested in the future by cell depletion approaches.

Chemotherapy with DOX in the PyMT alone was not sufficient to induce lasting tumor control. Our mouse model thus mimicked the situation in cancer patients, where its use as a single drug is hampered by tumor resistance. Drug resistance mechanisms have predominantly been tested in 2D or 3D cell culture (11). Therefore, transcriptomic data from our chemoresistance model comparing DOX therapy to the IgG1 control group might be of interest for future studies in this direction. There was a pattern of increased signaling through the Hedgehog pathway, through Ras and GPCRs such as sphingosine-1-phosphate receptors (S1PRs) upon treatment with DOX when chemoresistance was established. These signaling pathways were all prominently connected to tumor growth in the past (52–54), which may provide an explanation why chemotherapy in this model failed. Indeed, we recently described that blocking S1PR4 signaling improved chemotherapy response and prevented tumor relapse in the PyMT model (55).

Despite of the promising results combinatorial chemoimmunotherapy has shown, the individual clinical outcome for breast cancer patients remains difficult to predict. Our data reveal a gene signature with potential prognostic value. This gene signature consists of four genes that were upregulated in the DOX/anti-PD-1 group relative to the anti-PD-1 monotherapy group, namely *DIO2, PDK4, GSN*, and *MMP3*. Not all of the proteins were previously associated with a positive prognosis in cancer. The association of DIO2 overexpression in endometrial and colorectal cancer with a favorable prognosis (25) is in accordance with our findings in breast cancer. Also the observation that downregulation of GSN in breast cancer promoted malignant transformation (33) agrees with our study. PDK4 on the contrary was connected to poor patient outcome in breast cancer (28). This study utilized TCGA data as opposed to METABRIC data used in our study and a different cut-off strategy based on the number of cases designated as PDK4-positive. By simply dividing patients in upper and lower quartiles and using a database with more cases, we observed a positive correlation of PDK4 expression with survival in breast cancer patients. Also MMP3 expression was connected to promoting rather than restricting mammary carcinoma (36). This discrepancy to our study is not necessarily contradictory, since this study did not observe any differences in patient outcome regarding overall survival, but in distant metastasis-free survival (DMFS). Here, the prognostic value was also strongly dependent on tumor subtype and grade. It was stated that in HER2-positive tumors, such as PyMT tumors (13), an association of MMP3 expression with DMFS was not significant.

Clearly, studies investigating protein expression, activity, and cellular localization of these four markers in the tumor microenvironment are required to determine their precise impact on tumor development. It is important to note that the predictive value of our four gene signature was independent of treatment (hormone, radio-, or chemotherapy) in the METABRIC cohort. There was also no difference in the expression of the four genes irrespective of whether patients did or did not receive chemotherapy, while patients receiving hormone or radiotherapy actually expressed lower levels of these genes. Thus, the four gene signature predicts survival independent of prior standard of care treatment. It will be interesting to see how its expression is affected in patients receiving immune checkpoint blockade in the future. Importantly, to the best of our knowledge, an impact of these proteins in anti-tumor immunity or lymphocyte function has not been reported, indicating that the success of sensitizing for immune checkpoint blockade may be determined, at least partially, independently of a direct impact on cytotoxic lymphocytes.

## Data Availability Statement

The datasets presented in this study can be found in online repositories. The names of the repository/repositories and accession number(s) can be found below: NCBI under accession number GSE149479.

## Ethics Statement

The animal study was reviewed and approved by Hessian animal care and use committee were followed (approval numbers: FU1127 and FU1191).

## Author Contributions

ES-F, CO, and AW conceptualized and designed research. ES-F, AF, and AW developed methodology. ES-F, AF, CO, MB, and AH performed experiments and acquired data. ES-F, TS, and AW analyzed and interpreted results. KT and BB provided technical and material support. BB and AW supervised research and all authors participated in writing the manuscript. All authors contributed to the article and approved the submitted version.

## Conflict of Interest

KT was employed by ONO Pharmaceutical Co., LTD. The remaining authors declare that the research was conducted in the absence of any commercial or financial relationships that could be construed as a potential conflict of interest.
