# Supplementary material for: Immune Checkpoint Blockade Improves Chemotherapy in the PyMT Mammary Carcinoma Mouse Model
Source: Front Oncol. 2020 Sep 10;10:1771. doi: 10.3389/fonc.2020.01771 (PMC7513675; doi:10.3389/fonc.2020.01771)
Supplement: Supplementary file 3 [file Data_Sheet_1.PDF]

Supplementary Figures and Figure legends

Supplementary Figure 1

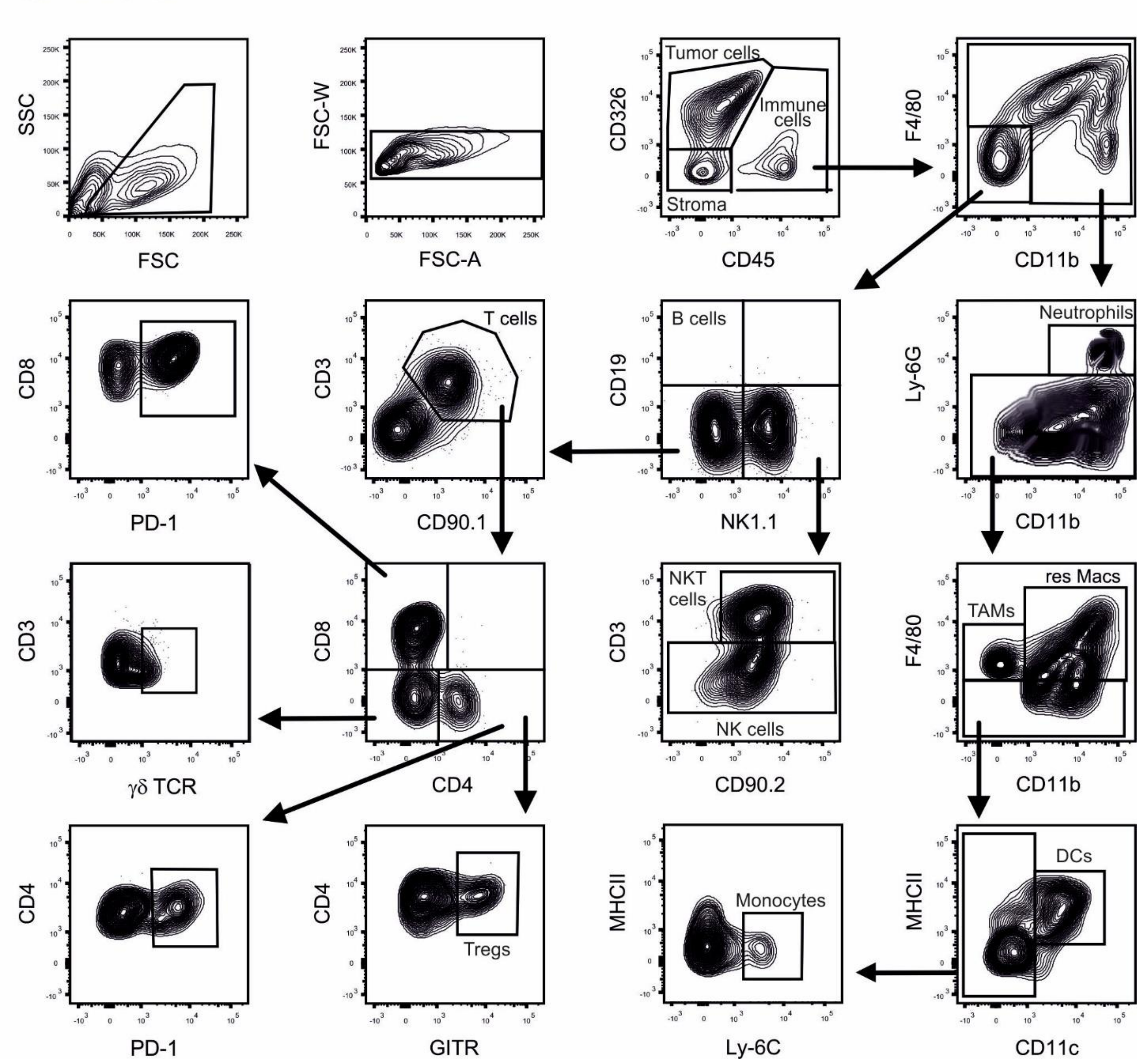

Supplementary Figure 1. Gating strategy for tumor immune cells. First, viable cells and cell doublets were discriminated by their SSC and FSC characteristics. Single cells were classified as tumor cells (CD326+ CD45-), stroma (CD326- CD45-) and immune cells (CD326- CD45+). Immune cells were further characterized as neutrophils (CD326- CD45+ Ly-6G+ CD11b+), tumor-associated macrophages (TAMs, CD326- CD45+ Ly-6G- F4/80+ CD11blow), resident macrophages (res Macs, CD326- CD45+ Ly-6G- F4/80+ CD11bhigh), DCs (CD326- CD45+ Ly-6G- F4/80- CD11b+ MHCII+ CD11c+), monocytes (CD326- CD45+ Ly-6G- F4/80- CD11b+ MHCII- CD11c- Ly-6C+), B cells (CD326- CD45+ F4/80- CD11b- CD19+), NK cells (CD326- CD45+ F4/80- CD11b- NK1.1+ CD3- CD90.2+/-), NKT cells (CD326- CD45+ F4/80- CD11b- NK1.1+ CD3+ CD90.2+), T cells (CD326- CD45+ F4/80- CD11b- CD19- NK1.1- CD90.2+ CD3+), CD4+ T cells (CD4+,

CD326- CD45+ F4/80- CD11b- CD19- NK1.1- CD90.2+ CD3+ CD4+), CD4+/PD-1+ T cells (CD4+/PD-1, + CD326- CD45+ F4/80- CD11b- CD19- NK1.1- CD90.2+ CD3+ CD4+ PD-1+), regulatory T cells (Tregs, CD326- CD45+ F4/80- CD11b- CD19- NK1.1- CD90.2+ CD3+ CD4+ GITR+), cytotoxic T cells (CD8+, CD326- CD45+ F4/80- CD11b- CD19- NK1.1- CD90.2+ CD3+ CD8+), CD8+/PD-1+ T cells (CD8+/PD-1+, CD326- CD45+ F4/80- CD11b- CD19- NK1.1- CD90.2+ CD3+ CD8+ PD-1+), and γδ T cells (CD326- CD45+ F4/80- CD11b- CD19- NK1.1- CD90.2+ CD3+ CD4- CD8- γδ TCR+). Representative contour plots from an IgG1 treated PyMT tumor are shown.

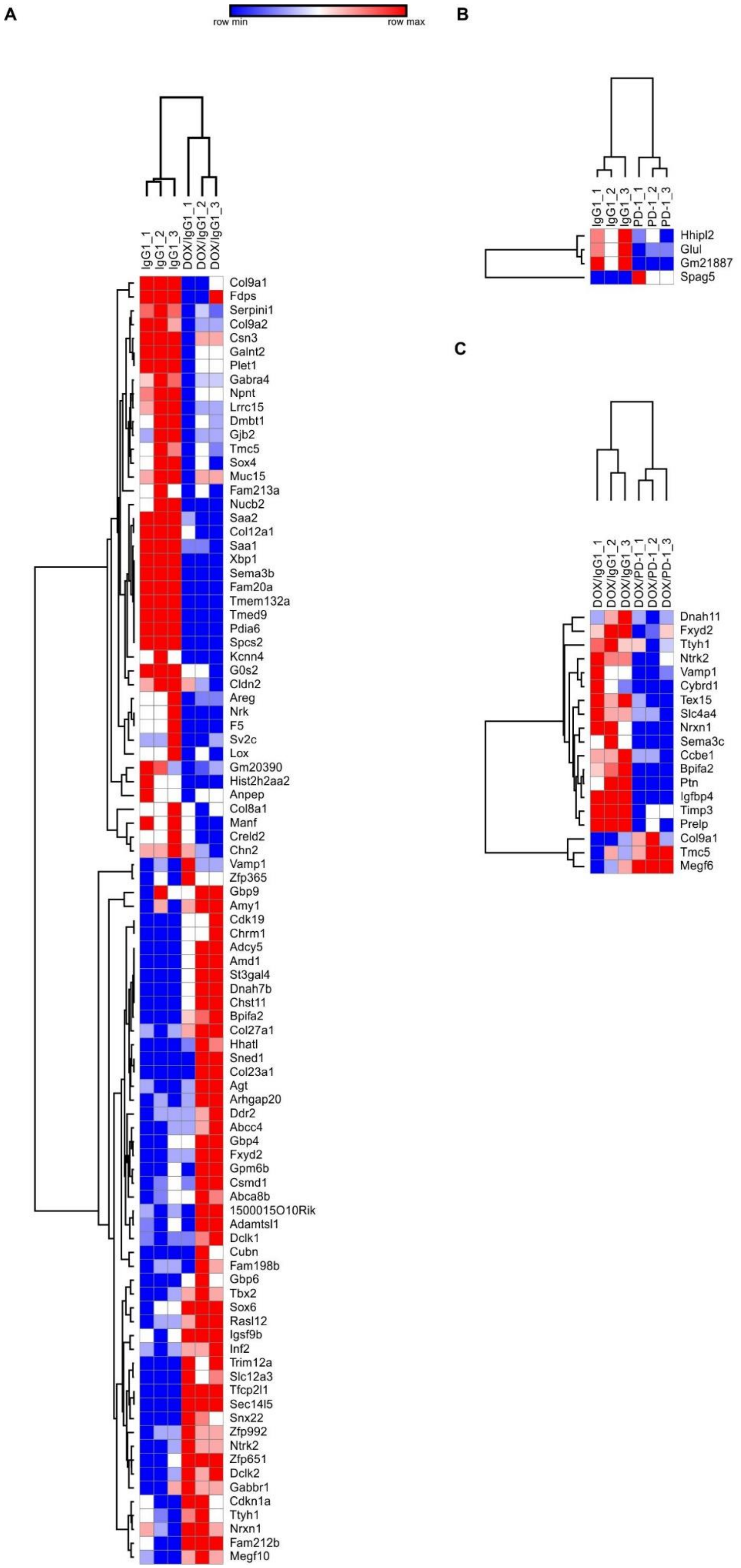
A
row min
row max
IgG1_1
IgG1_2
IgG1_3
DOX/IgG1_1
DOX/IgG1_2
DOX/IgG1_3
Col9a1
Fdps
Serpini1
Col9a2
Csn3
Galnt2
Plet1
Gabra4
Npnt
Lrrc15
Dmbt1
Gjb2
Tmc5
Sox4
Muc15
Fam213a
Nucb2
Saa2
Col12a1
Saa1
Xbp1
Sema3b
Fam20a
Tmem132a
Tmed9
Pdia6
Spcs2
Kcnn4
G0s2
Cldn2
Areg
Nrk
F5
Sv2c
Lox
Gm20390
Hist2h2aa2
Anpep
Col8a1
Manf
Creld2
Chn2
Vamp1
Zfp365
Gbp9
Amy1
Cdk19
Chrm1
Adcy5
Amd1
St3gal4
Dnah7b
Chst11
Bpifa2
Col27a1
Hhatl
Sned1
Col23a1
Agt
Arhgap20
Ddr2
Abcc4
Gbp4
Fxyd2
Gpm6b
Csmd1
Abca8b
1500015O10Rik
Adamtsl1
Dclk1
Cubn
Fam198b
Gbp6
Tbx2
Sox6
Rasl12
Igsf9b
Inf2
Trim12a
Slc12a3
Tfcp2l1
Sec14l5
Snx22
Zfp992
Ntrk2
Zfp651
Dclk2
Gabbr1
Cdkn1a
Ttyh1
Nrxn1
Fam212b
Megf10
B
IgG1_1
IgG1_2
IgG1_3
PD-1_1
PD-1_2
PD-1_3
Hhipl2
Glul
Gm21887
Spag5
C
DOX/IgG1_1
DOX/IgG1_2
DOX/IgG1_3
DOX/PD-1_1
DOX/PD-1_2
DOX/PD-1_3
Dnah11
Fxyd2
Ttyh1
Ntrk2
Vamp1
Cybrd1
Tex15
Slc4a4
Nrxn1
Sema3c
Ccbe1
Bpifa2
Ptn
Igfbp4
Timp3
Prelp
Col9a1
Tmc5
Megf6

Supplementary Figure 2. Differentially expressed genes dependent on treatment. The heat maps show differentially expressed genes between PyMT tumors treated with IgG1 or doxorubicin (DOX) treatment (**A**), treated with IgG1 or PD-1 (**B**), or treated with DOX or DOX plus anti-PD-1 (**C**) (n = 3 each).

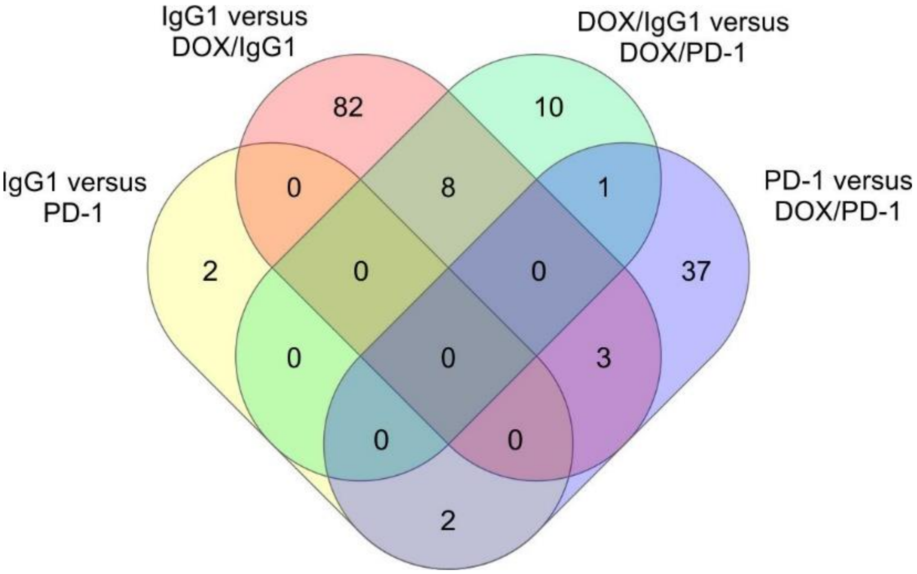

Supplementary Figure 3. Overlap between gene signatures. Gene signatures in Figure 3A and Figure S2 were compared for overlapping genes, which are indicated in the Venn diagram.
